# An ontology-based search engine for digital reconstructions of neuronal morphology

**DOI:** 10.1007/s40708-017-0062-x

**Published:** 2017-03-23

**Authors:** Sridevi Polavaram, Giorgio A. Ascoli

**Affiliations:** Center for Neural Informatics, Structures and Plasticity, Krasnow Institute for Advanced Study, George Mason University, Fairfax, VA USA

**Keywords:** Data mining, Ontological hierarchies, Metadata mapping, Concept-based searching, Semantic relations, Information retrieval

## Abstract

Neuronal morphology is extremely diverse across and within animal species, developmental stages, brain regions, and cell types. This diversity is functionally important because neuronal structure strongly affects synaptic integration, spiking dynamics, and network connectivity. Digital reconstructions of axonal and dendritic arbors are thus essential to quantify and model information processing in the nervous system. NeuroMorpho.Org is an established repository containing tens of thousands of digitally reconstructed neurons shared by several hundred laboratories worldwide. Each neuron is annotated with specific metadata based on the published references and additional details provided by data owners. The number of represented metadata concepts has grown over the years in parallel with the increase of available data. Until now, however, the lack of standardized terminologies and of an adequately structured metadata schema limited the effectiveness of user searches. Here we present a new organization of NeuroMorpho.Org metadata grounded on a set of interconnected hierarchies focusing on the main dimensions of animal species, anatomical regions, and cell types. We have comprehensively mapped each metadata term in NeuroMorpho.Org to this formal ontology, explicitly resolving all ambiguities caused by synonymy and homonymy. Leveraging this consistent framework, we introduce OntoSearch, a powerful functionality that seamlessly enables retrieval of morphological data based on expert knowledge and logical inferences through an intuitive string-based user interface with auto-complete capability. In addition to returning the data directly matching the search criteria, OntoSearch also identifies a pool of possible hits by taking into consideration incomplete metadata annotation.

## Introduction

As neuroscience transitions into the Big Data era, neuroinformatics resources, such as databases, search engines, and web services are playing an ever more central role [1]. The continuous growth in the number and size of digitally available datasets already offers considerable research opportunities. Data sharing, however, can only achieve its full potential if the accompanying metadata are also machine-readable. This is particularly important in neuroscience in light of the absence of standardized terminologies [2], leading to the constant need of domain expertise to reconcile confusing, conflicting, or inconsistent nomenclatures. One publication might report the molecular profile of a ‘chandelier cell’ from the ‘primary visual area’ of ‘rhesus monkeys’; a second article in the same issue of the journal might quantify the electrophysiological properties of an ‘axo-axonic interneuron’ from the ‘occipital cortex’ of ‘macaca mulatta.’ A computer (and most human readers) would have a hard time realizing that the two papers are referring to exactly the same neuron type, brain region, and animal species. At the same time, different authors might describe subjects of a given age as ‘young’ or ‘adult,’ due to either disagreement on the definition or sharply distinct experimental contexts.

**Fig. 1:**
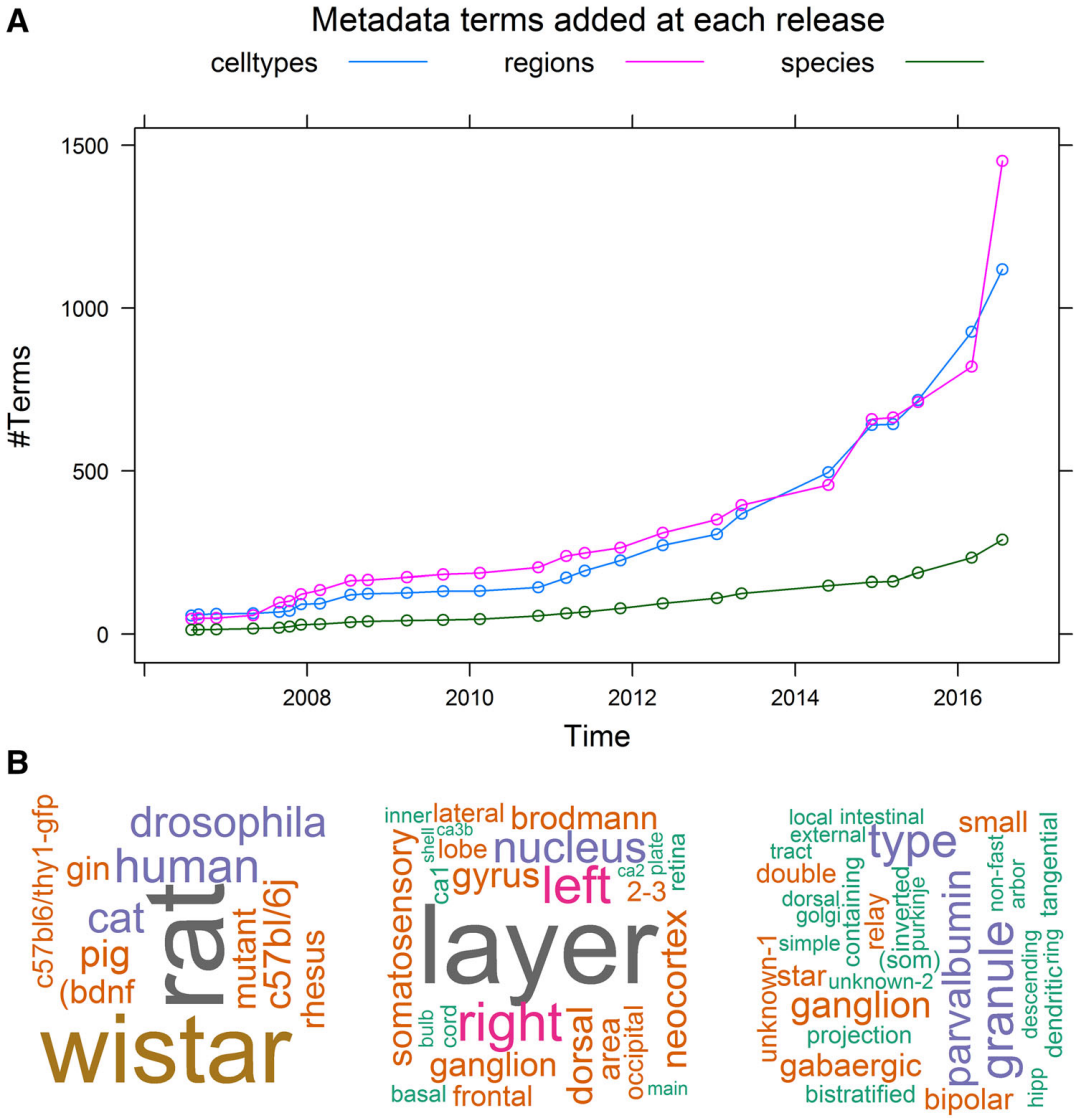
Metadata complexity. **a** Temporal trend in the counts of metadata terms used in NeuroMorpho.Org to annotate the animal species (including strains), brain regions, and cell types. Each data point marks a version release. **b** Word clouds of the most common terms in the database across the main categories (from *left* to *right*) of animal species/strains, brain regions, and cell types. Word size corresponds to relative usage frequency

Digital reconstructions of neuronal morphology provide a particularly relevant case in point [3, 4]. A rich selection of electronic tools for tracing, visualizing, analyzing, and modeling axonal and dendritic arbors supports a vibrant computational neuroanatomy community [5]. Between one-third and one-half of the authors of tracing studies agree to share their data, and all available reconstructions are freely available online in the public repository NeuroMorpho.Org [6]. The number of digitally traced neurons in NeuroMorpho.Org grew from 1000 in the first open release in 2006 to over 50,000 in version 7.0 10 years later. Every entry in the database is annotated with specific details regarding the animal subject, anatomical region, cell type, as well as the completeness of data content and the most relevant information about the experimental preparation, including histological, imaging, and reconstruction protocols [7]. Although metadata annotation in NeuroMorpho.Org is human-curated, the conceptual descriptors largely reflect the authors’ selections (Fig. 1). As a consequence, the number and variety of distinct terms grows with the amount of data at every version release (Fig. 1a). On the one hand, this trend forces users of the repository to cope with a progressively more complex vocabulary (Fig. 1b). On the other, it also aggravates the curators’ task of annotating new datasets, due to the increasing overhead of consistency checks and required corrections of terminological ambiguities, redundancies, and discrepancies.

In order to alleviate these difficulties, we have comprehensively re-organized the metadata of NeuroMorpho.Org into *ontological hierarchies*. Scientific knowledge is often conceptualized hierarchically [8]. Perhaps the most famous example in biology is the comprehensive taxonomical representation of living organism phylogeny [9]. Other more specific domains that are conveniently described in conceptual hierarchies include the functional neuroanatomy of cerebral cortex [10], behavioral responses to sensory stimuli [11], and data structures in artificial intelligence [12].

Ontologies are formal representations of conceptual knowledge (notably including hierarchies) as rigorously consistent and machine-readable semantic structures [13] supporting powerful logic-based queries [14]. Ontologies are increasingly common in the biosciences [15]. Within the open biomedical ontologies (OBO) umbrella, gene ontology (GO) is widely used for annotating genome sequences and their functions, and parallel efforts exist in anatomy [16]. Ontologies are gaining popularity in neuroscience as well [17–20]. For example, the fly community produced the user-friendly web-based interface virtualflybrain.org harnessing controlled vocabularies and other advanced functionalities [25].

Here we present the design and implementation of ontologies applied to the novel hierarchical organization of NeuroMorpho.Org metadata, providing a practical solution for neuromorphological data management and information retrieval. Formal ontological mapping of all metadata terms ensured logical validation of the underlying concepts while enabling the addition of semantic relations. This article also introduces a powerful novel functionality for querying NeuroMorpho.Org data based on inferential reasoning. Lastly, we discuss challenges and opportunities related to the long-term maintenance, expansion, and sustainability of hierarchy-based annotation using controlled vocabularies.

## OntoSearch: design and implementation

**Fig. 2:**
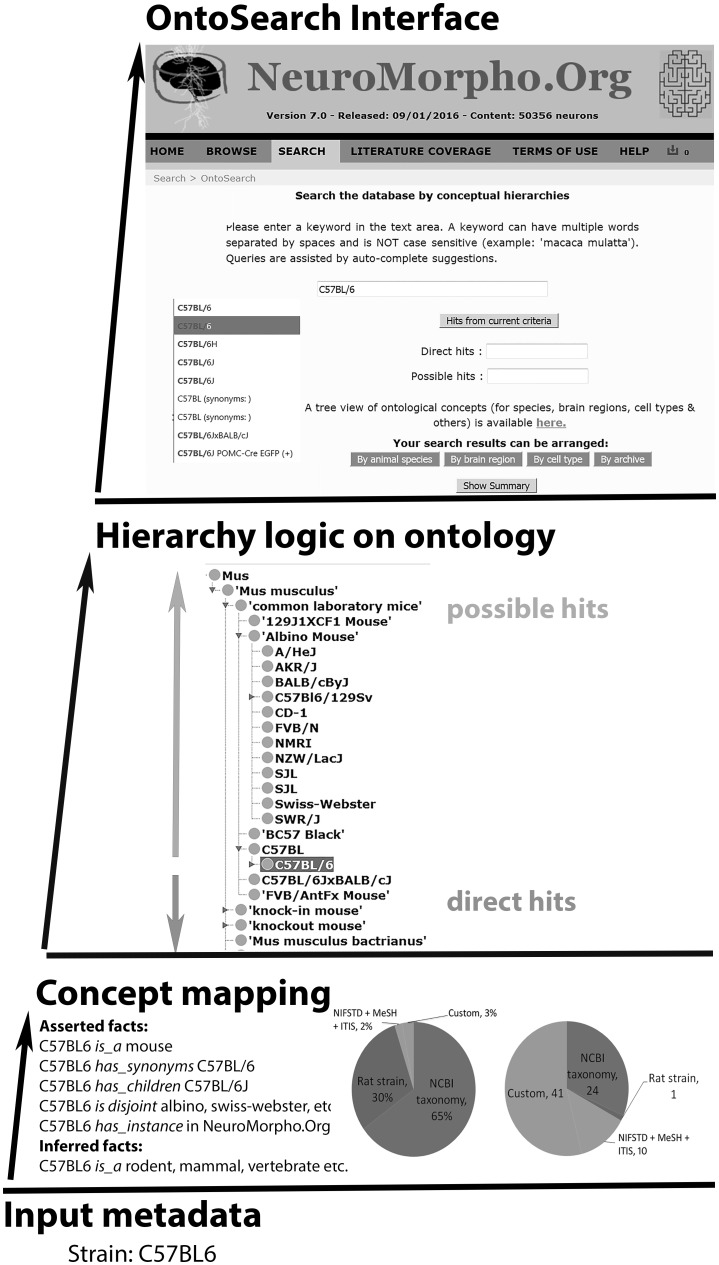
OntoSearch framework. A metadata string transforms into a uniquely identified concept with concept mapping, as identified by the associated facts. The mapped concept gets integrated into the ontology enabling retrieval of possible hits and direct hits by crawling up and down the hierarchy. The search results are displayed on the OntoSearch interface facilitated by auto-complete feature allowing the user to browse/download the results

The OntoSearch engine application relies on two related yet distinct processes: hierarchical metadata organization and formal ontological model (Fig. 2). These processes are described in more detail in the following sub-sections. Briefly, the metadata hierarchies constitute the backbone of the knowledge structure, and all properties previously annotated in NeuroMorpho.Org were re-mapped to the appropriate concept nodes. The ontological model adds inferential relationships to the hierarchies and defines the reasoning logic to identify the database entries (neuronal reconstructions) concordant to a given query. The related web-based graphical interface, fully integrated in NeuroMorpho.Org v.7.0, enables user-friendly queries and data retrieval.

### Modeling metadata as conceptual hierarchies

**Fig. 3:**
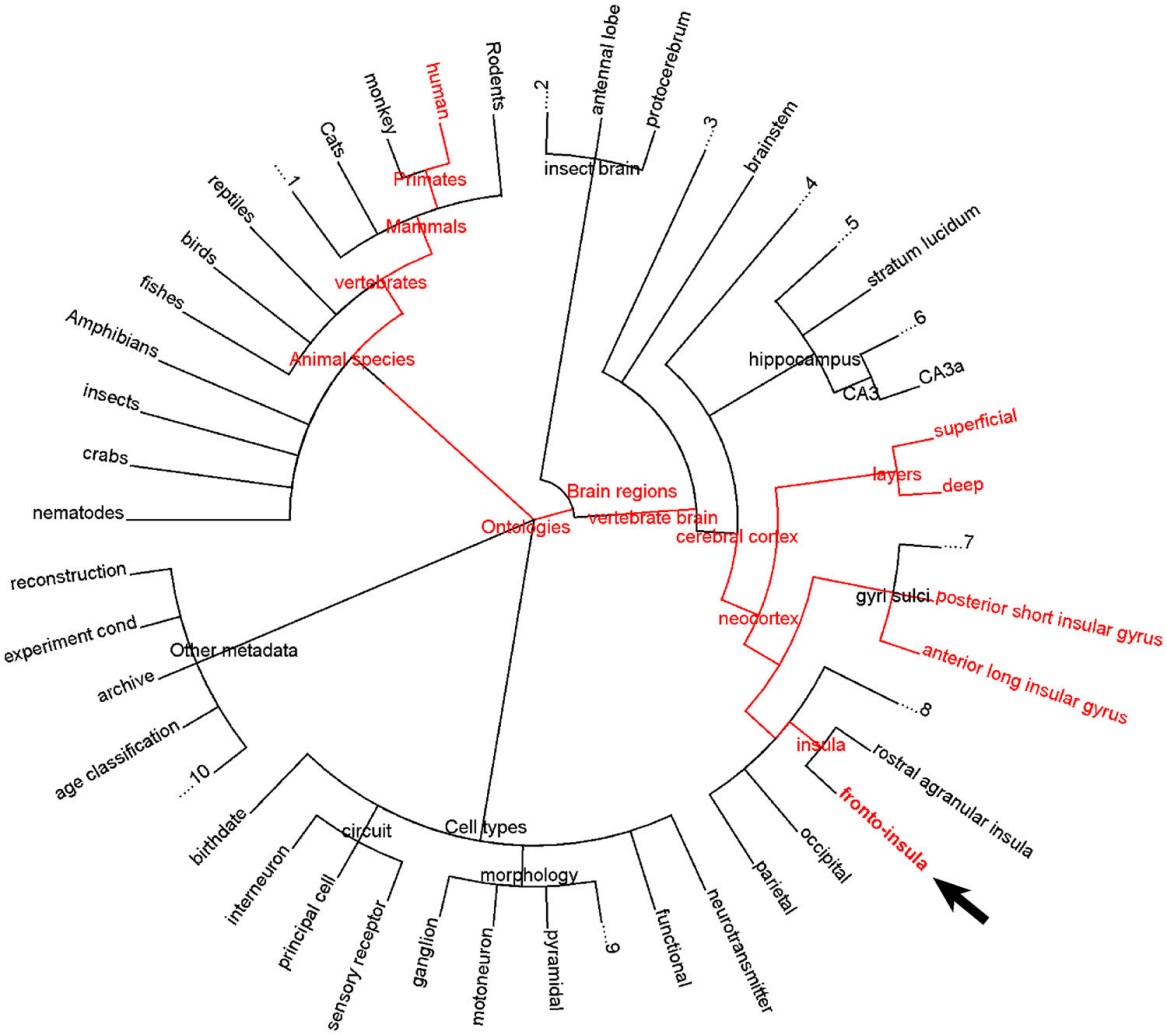
NeuroMorpho.Org metadata dimensions. The annotation of neuronal reconstructions follows the ontological classification as shown here for species, brain regions, cell types and other metadata. All four dimensions are partially expanded into independent hierarchies where each concept has at least one matching instance in NeuroMorpho.Org. The *red* highlight exemplifies the brain region ‘fronto-insula’ being hooked to ‘humans’ in species, thereby connecting all concepts except the disjoint ones along the path. (Color figure online)

The NeuroMorpho.Org ontology is built upon a set of hierarchies corresponding to the main metadata dimensions: animal species strains, anatomical region, neuron type, and other properties (Fig. 3). Some dimensions (such as species) are described by a single hierarchy; others (such as anatomical regions) require multiple hierarchies. For instance, the mammalian neocortex is commonly organized in at least two orthogonal directions, one describing the surface position often identified by a functional domain (e.g., primary somatosensory, left hind limb), and the other describing laminar depth typically referring to cytoarchitecture and microcircuitry (e.g., layer 5b). The logical relationships among hierarchies within and across metadata domains are described in Sect. 2.2.

Every hierarchy is composed of a set of nodes corresponding to unique concepts. When multiple strings refer to the same concepts, one is selected as the preferred term and the others are listed as *synonyms*. Every node except the root of the hierarchy (which is orphan) has exactly one parent and may have one or more children. Children are linked to parents with a *subsumption* relationship (‘*is_a*’ or ‘*is_part_of*’), meaning that all properties of a parent apply to all of its children; for instance, if rodents have four legs and a rat *is_a* rodent, then rats must have four legs as well. Furthermore, sibling concepts are mutually exclusive: an animal cannot be at the same time a mouse and a rat.

Hierarchies were assembled leveraging as much as possible machine-readable knowledge from existing resources. Since the goal was to map string-based annotation of properties of NeuroMorpho.Org reconstructions, we simplified the source hierarchies by pruning off all the descendant branches and sub-trees with no correspondence in the available data. However, since we always preserved the entire ancestor lineage of each mapped concept up to the root, each hierarchy typically includes many relevant nodes that were not explicitly annotated for all neuronal tracings. For instance, ‘rodent’ and ‘mammal’ are included even though these concepts had not been explicitly indicated for any of the relevant reconstructions. Less frequently, we also had to add new nodes when required concepts were missing from available ontologies (see following paragraphs for examples). The main metadata dimensions are each described below and summarized in Table 1 at the end of Sect. 2.1. The complete hierarchies are available as developer version: https://bioportal.bioontology.org/ontologies/NMOBR?p=classes and visualization version: https://bioportal.bioontology.org/ontologies/NMOBR_2?p=classes.

**Table 1:** Numerical summary of NeuroMorpho.Org metadata (v7.0) hierarchical organization

Metric/dimension	Animal species	Brain regions	Cell types	Other properties
# Hierarchies	1	13	10	8
# Concepts	1488	401	282	412
# Mapped preferred terms	156	390	287	393
# Synonyms	550	199	45	8

#### Species hierarchy

Animal species and strains are represented by a single *is_a* hierarchy (where parents and children are *hypernym* and *hyponyms*, respectively) modeled after the widely accepted phylogenetic organization of the tree of life [9]. A vast majority (>80%) of these concepts are imported from the National Center for Biotechnology Information (NCBI) animal kingdom taxonomy [21, 22]. At the strain level, we did not follow strict genetic lineage to optimize practical usability according to broad neuroscience usage. For example, several mice strains lacking pigmentation are broadly grouped under ‘albino,’ others that are genetically engineered are grouped as ‘transgenic,’ while mutant strains with targeted gene insertion or deletion are grouped as ‘knock-in’ or ‘knockout,’ respectively. Custom-added strains (see, e.g., those of Rattus norvegicus in Fig. 3) were imported from NIF-Organism (http://ontology.neuinfo.org/NIF/BiomaterialEntities/NIF-Organism.owl#), Rat Genome Database (http://rgd.mcw.edu/rgdweb/ontology/search.html?term=RS%3A0000457&ont=RS), Jackson lab (https://www.jax.org/mouse-search), and flybase.org. The species hierarchy constitutes the richest, most mature, and best organized metadata dimension in NeuroMorpho.Org. Due to the depth of this knowledge base, the number of concepts in this hierarchy exceeds by a full order of magnitude that of explicitly annotated metadata terms (Table 1).

#### Anatomical regions

Anatomical regions can be described in terms of *is_part_of* relationships, where parents and children are *holonyms* and *meronyms*, respectively. Since the nervous system is embedded in physical space, it is natural to envision three perpendicular dimensions to represent somatic locations. The choice of the axial directions, however, is not unique even within a given species and neural structure. Depending on the experimental design, for example, different studies could report the same position in hippocampal area CA1 relative to the canonical brain axes (dorso-ventral, rostro-caudal, medio-lateral) or from the internal perspectives of the hippocampus (septo-temporal, proximo-distal, superficial-deep). Furthermore, it is often scientifically sensible, practically convenient or simply customary to adopt complementary cytoarchitectonic, developmental or functional parcellations instead of Cartesian coordinates [23]. Last but not least, the nervous systems of different animal species such as nematodes, fruit flies, zebrafish, mice, and humans have vastly different anatomical organizations. As a result of a combination of the above factors, as many as 13 distinct hierarchies were required to map the positional annotation of traced neurons available in NeuroMorpho.Org. Due to lack of agreed-upon consensus in the community and the relative sparsity of machine-readable anatomical knowledge across several of the needed dimensions, the NeuroMorpho.Org brain region hierarchies were assembled from a number of external references. The vertebrate hierarchy incorporates the layered architecture of the cerebral cortex and the mouse neocortical parcellation of the Allen Brain Atlas [24]. The anatomical, functional, and developmental classification of primate brain regions is sourced from BrainInfo.org [23]. Fly neuropil is adapted from the VirtualFly.org [25] and standard insect nomenclatures [26]. The *Caenorhabditis elegans* anatomy follows the WormBase.org atlas [27].

#### Cell types

Neuron classification is still a controversial topic [28, 29], and the few available machine-readable resources [18, 30–32] are only sparsely populated relative to the diversity of content in NeuroMorpho.Org. Our best attempt to organize, integrate, and map the existing annotation on suitable knowledge yielded ten cell type hierarchies spanning the morphology, neurotransmitter, development (birthday), molecular biomarker, electrophysiology, circuitry (e.g., ‘local’ vs. ‘projecting’), and functional dimensions. Unsurprisingly, the main morphological hierarchy provides the richest mapping of reconstruction data, listing over 120 distinct neuron types including numerous canonical morphologies [20] such as Cajal–Retzius, chandelier, Martinotti, mitral, Purkinje, Renshaw, and von Economo cells. For the most represented neuron type in NeuroMorpho.Org, the pyramidal cell, in addition to several morphological sub-types in the main hierarchy (e.g., Betz, oblique, and star-pyramidal), a few supplementary conceptual dimensions were required to describe complementary properties (e.g., early- vs. late-bifurcating, thick- vs. slender-tufted).

#### Other properties

All the other dimensions that can be transformed into independent hierarchies are grouped under ‘Other metadata.’ These concepts are organized into eight ontological hierarchies (Table 1) representing a total of 412 concepts in the following metadata: ‘experimental condition,’ ‘objective type,’ ‘age classification,’ ‘archive,’ ‘protocol design,’ ‘reconstruction method,’ ‘slicing direction,’ and ‘staining method.’ Although these dimensions are mostly ‘flat,’ they nonetheless expand the search vocabulary (393 mapped terms and eight synonyms).

### Ontological model and web-based search functionality

The NeuroMorpho.Org ontology links concepts using three types of logical relationships: subsumption, equivalence, and implication. As explained above, the many-to-one subsumption relations ‘*is_a*’ and ‘*is_part_of*’ constitute the fundamental connectors in single-parent hierarchies (e.g., ‘GIN mouse’ *is_a* ‘transgenic mouse’; ‘CA1’ *is_part_of* ‘hippocampus’). The equivalence relation identifies the same concepts across distinct hierarchies within a single dimension. For instance, the hippocampus can be conceptually partitioned into sub-regions (e.g., CA3, CA1) or in layers (e.g., stratum oriens, stratum radiatum) in two parallel hierarchies within the brain region dimension. Defining the equivalence of the hippocampus concept in these hierarchical alternatives effectively connects the two categorizations schemes at a common node. Lastly, the implication relation ‘hooks’ concepts between hierarchies within or across knowledge dimensions based on specific biological constraints. As an example of implication that logically connects separate dimensions, the ‘Purkinje cell’ concept is hooked to ‘Cerebellum,’ because these neuron types are exclusively found in that brain region. Altogether, the NeuroMorpho.Org metadata ontology contains 2818 subsumptions, 12 equivalence relations, and 221 implications (hooks).

**Fig. 4:**
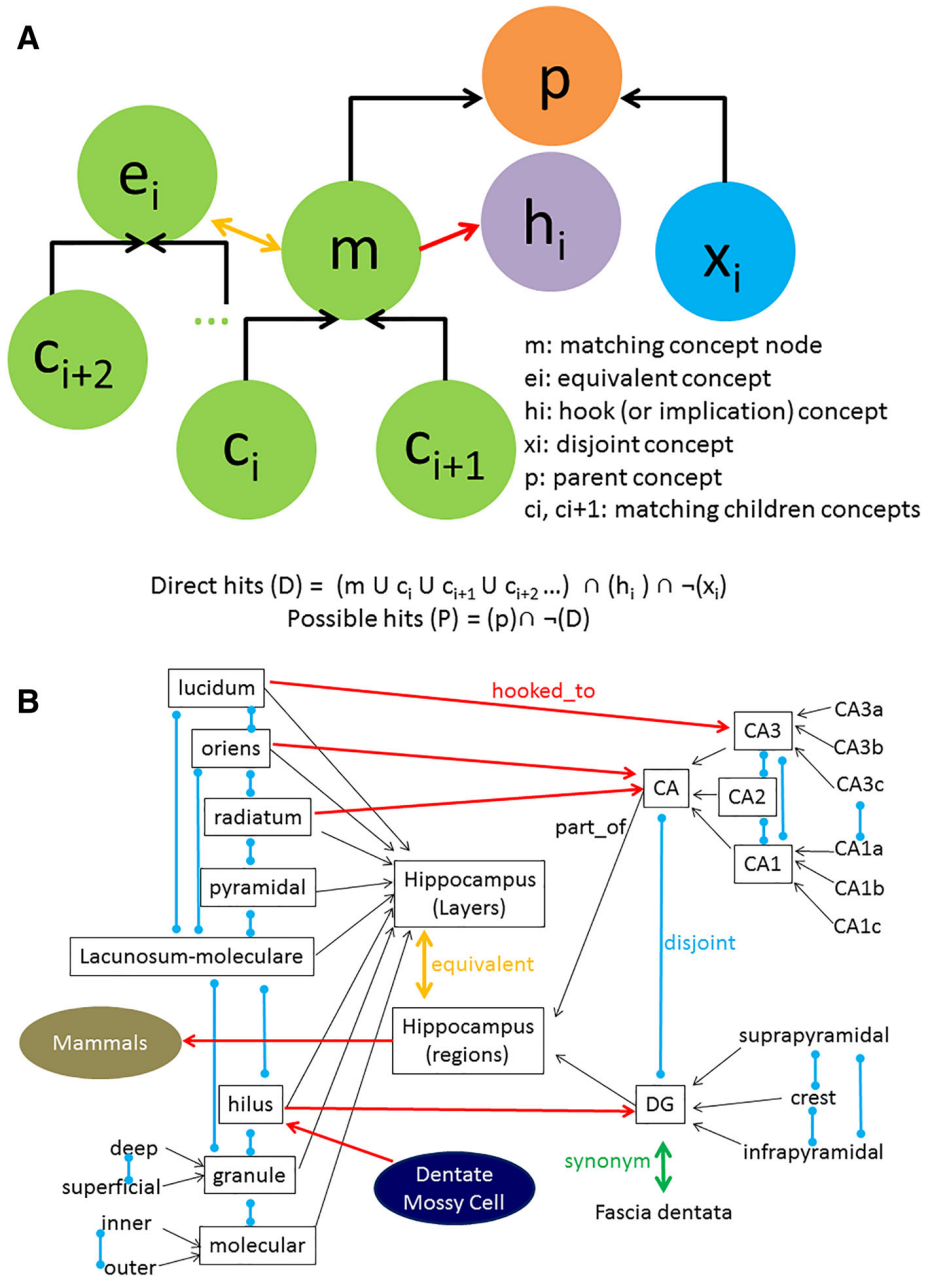
Logical relations. **a** Schematic of the ontological links between concepts within hierarchy (*black arrows*) and across hierarchies (*red arrows*). The OntoSearch algorithm traverses the nodes to find direct hits (*green*) and possible hits (*orange*). The hooks (*purple*), equivalent concepts (*green*), and disjoint nodes (*blue*) also influence the equation for direct hits (*D*) and possible hits (*P*). **b** Subset of logical relations among hippocampus regions and layers. Not all links are displayed for the sake of display clarity. (Color figure online)

The OntoSearch functionality (the implementation of which is described below) leverages these three logical relationships to find the concepts matching a given query and to retrieve the corresponding instances (reconstructions). Specifically, the engine searches the pool of hierarchies for ‘direct’ and ‘possible’ hits (Fig. 4). Direct hits are concepts that have the certainty to match the search term, and include all synonyms of the target node plus all of its descendants. For instance, searching for ‘rattus’ will retrieve neurons from rat (synonym) as well as Wistar, Fischer, and Sprague–Dawley (children). OntoSearch finds the direct hits by crawling down each hierarchy from the target node. Possible hits in contrast are concepts that might match the search term given the available annotation, but cannot be ascertained for sure. Consider the example of a set of rat neurons for which the authors did not report the specific strain. A search for ‘Wistar’ will identify these neurons as possible hits, but will exclude all neurons known to be from Sprague–Dawley animals (disjoint sibling). OntoSearch finds the possible hits by crawling up the hierarchy for instances corresponding to ancestor concepts that are not specified to match any disjoint sibling of the target search.

When crawling up and down the hierarchies to identify direct and possible hits, OntoSearch also traverses equivalent and hooked nodes, thus transforming a single target concept into a conjunctive query (Fig. 4a). For example, searching for ‘stratum lucidum’ identifies as possible hits any neuron in CA3 that is not specified to be located in other layers; because of the equivalence of the CA3 concept between layers and sub-areas, neurons specified to be located in CA3a, CA3b or CA3c will also be matched as possible hits if their layer is not indicated (Fig. 4b). Similarly, searching for Purkinje will return as possible hits any cerebellar neurons (because of the ‘hooked’ implication) that are not specified to be of a different cell type.

**Fig. 5:**
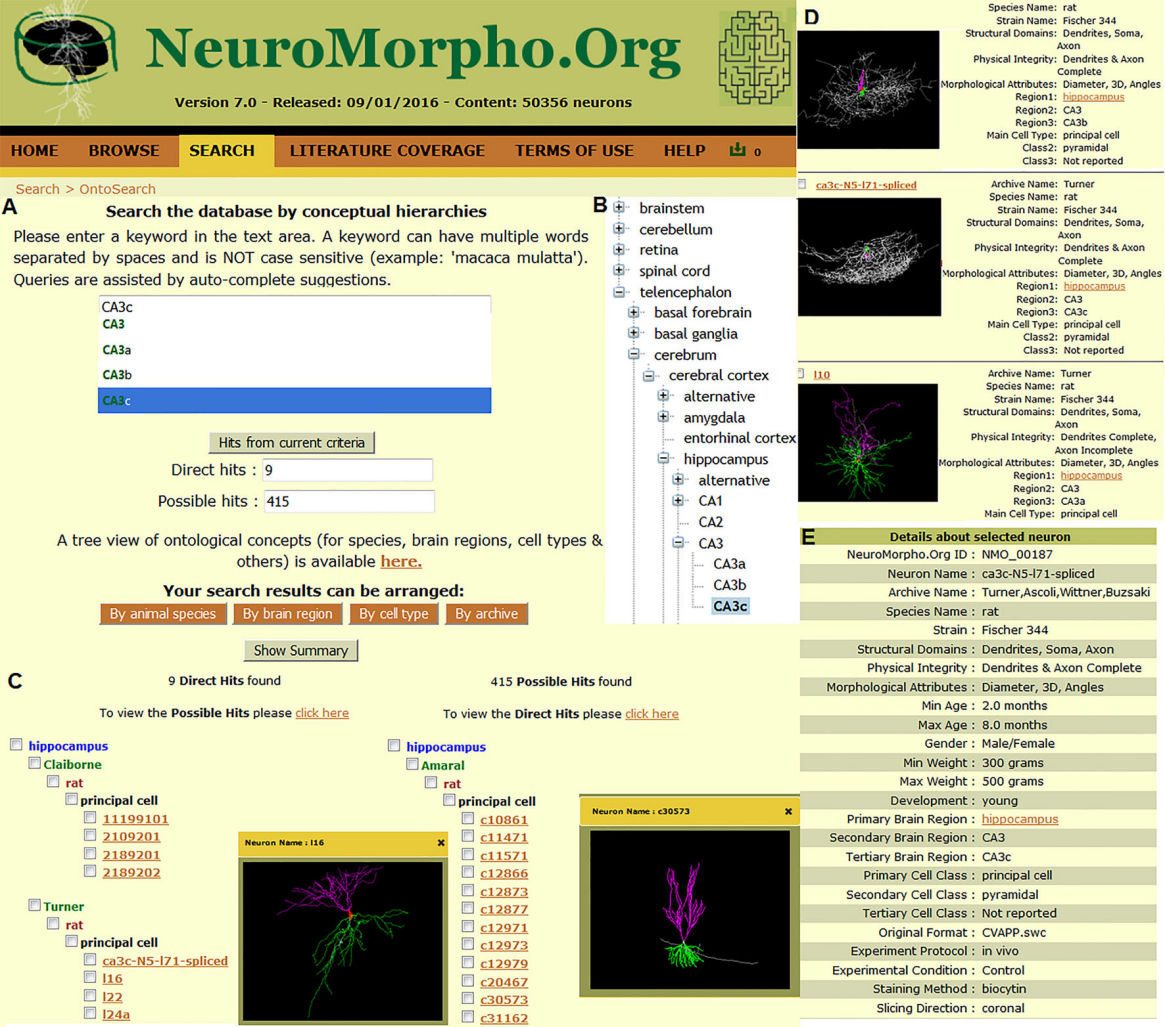
OntoSearch interface. **a** The direct and possible hits on the OntoSearch interface, **b** Corresponding hierarchical ontology lexicon, **c** the browse ‘by region’ view of the nine direct hits and the 415 possible hits, **d** the ‘show summary’ view of the direct and possible hits, **e** and detailed view of the selected neuron are provided as end result of the logic

OntoSearch harnesses the hierarchical logic and semantic reasoning described above to provide powerful yet intuitive data mining capabilities. Users can search for data from a simple text-based query interface (NeuroMorpho.Org/OntoSearch.jsp). The search bar suggests suitable term selections through auto-completion of typed strings (Fig. 5). The auto-complete vocabulary is sourced by 3429 preferred names and synonyms, more than tripling the original metadata annotation of NeuroMorpho.Org. The results are returned in two separate sets of direct and possible hits. Importantly, this web-based search functionality is fully integrated in the NeuroMorpho.Org computational infrastructure (Fig. 5a–e). Thus, as in customary browsing mode, users can group the search results by specific dimensions (species, brain regions, neuron types or contributing laboratories) or, alternatively, display them as summary lists. Identified neurons can be downloaded in bulk or individually, while selecting any neuron names opens the detailed view of the corresponding data, metadata, and morphometric measurements.

Metadata hierarchies and logical relationships were implemented in Web Ontology Language (OWL) and are available open-source (https://bioportal.bioontology.org/ontologies/NMOBR). In OWL, a *concept* is an entity (or *class*) labeled by a unique identifier and defined by a combination of annotation properties, relationships, and instances. Namespace is the domain under which a concept is published. The OWL naming syntax (e.g., github.com/jamesaoverton/obo-tutorial/blob/master/docs/names.md) requires that all namespaces end with #, /, or _. For example, the concept *rTg4510 P301*-*L mutant* is from the NeuroMorpho.Org species ontology namespace *NMOSp.owl#*. The international resource identifier (IRI) functions as a unique reference of the concepts, while CURIE is an abridged form of IRI that avoids listing long and redundant identifiers. The IRI and CURIE for the above-mentioned strain are, respectively, neuromorpho.org/ontologies/NMOSp.owl#NMOSp_1057 and NMOSP:NMOSp_1057. Table 2 lists additional examples of concepts, their IRIs and CURIEs.

**Table 2:** Examples of NeuroMorpho.Org concepts, unique identifiers, and short forms

Concept	Unique identifier (IRI)	Short form (CURIE)
Transgenic mouse	http://id.nlm.nih.gov/mesh/D008822	Mesh:D008822
GIN mouse	http://neuromorpho.org/ontologies/NMOSp.owl#NMOSp_1040	NMOSp:NMOSp_1040
*Mus musculus*	http://purl.obolibrary.org/obo/NCBITaxon_10090	obo:NCBITaxon_10090
Hippocampus	http://neuromorpho.org/ontologies/vertebrateH.owl#NMOBr_392	vertebrateH:NMOBr_392
Hippocampus (layer-equiv.)	http://neuromorpho.org/ontologies/hiplayerH.owl#NMOBr_392	hiplayerH:NMOBr_392
Stratum radiatum	http://neuromorpho.org/ontologies/hiplayerH.owl#NMOBr_394	hiplayerH:NMOBr_394
CA1	http://neuromorpho.org/ontologies/vertebrateH.owl#NMOBr_139	vertebrateH:NMOBr_139

OWL applies naming standards to annotation properties. For all NeuroMorpho.Org ontologies, we use the *rdfs:label*, *oboInOwl:hasExactSynonym*, and *OboInOwl:hasDBXref* to store the preferred name, synonyms, and references, respectively. Use of OntoMaton [33] and OBO-edit [34] alleviates the manual process of mapping instances to matching concept and finding references. Custom Java code converts tabbed hierarchies into a template of triples, which is then run through open-source tools (github.com/ontodev/robot) to produce OWL/XML format ontologies. Using the concept CURIEs of Table 2, subsumption is expressed in triple notation as <NMOSP:NMOSp_1040 *rdfs:subclassof* obo:NCBITaxon_10090>; equivalence as <vertebrateH:NMOBr_139 *equivalentClass* hiplayerH:NMOBr_139>; and implication as <vertebrateH:NMOBr_394 *vertebrateH:hasHook* hiplayerH:NMOBr_139>.

From the software design perspective, OntoSearch is run on the middle (‘knowledge’) layer between the user interface and the relational database. The translation of OWL reasoning inferences into SQL queries is implemented in Java using OWLAPI [35, 36] and Elk reasoner (https://github.com/liveontologies/elk-reasoner). Use of the OWL/XML format makes it possible to export the structured knowledge into other machine-readable formats such as the web standard for data interchange JSON.

## Querying NeuroMorpho.Org with OntoSearch

Here we present illustrative scenarios that demonstrate the use of OntoSearch to find neuromorphological data of interest from NeuroMorpho.Org v7.0 (Table 3).

**Table 3:** Representative use cases of queries and corresponding results

Search term	Direct hits	Exact matching concepts	Possible hits	Potential matching concepts
Rodents	17,903	{Mouse} U {rat} U {agouti} …	–	–
Vertebrates	22,820	{Rabbit} U {elephant} U {whales} U {rodents} U {carnivores} …	–	–
C57BL6 mouse	2309	{**C57BL/6**} U {C57BL/6J}	39	{C57BL} U {unspecified mouse}
Eastern tiger salamander	–	–	62	{Tiger salamander}
Philippine long-tailed macaque	–	–	191	{*Macaca fascicularis*}
Transgenic mouse	1914	{5HT3-EGFP} U {B13} U {Atoh1/nGFP} …	35	{Unspecified mouse}
Knock-in mouse	214	{Arx(GCG)7–1 JI (B6)} U {BDNF WT} U {BDNF Met/Met} …	5	{Unspecified mouse}
Knockout	161	{Bassoon (bsn) mutant} U {Ddo –/–} …	35	{Unspecified mouse}
CA1/CA2 border	3	{**CA1c**} ∩ {SP} ∩ {SO} ∩ {SR} ∩ {SLM} ∩ {mammals}	998	{CA1} U {hippocampus}
Hilus	40	{**Hilus**} ∩ {DG} ∩ {mammals}	407	{DG} U {hippocampus}
Stratum lucidum	16	{CA3} ∩ {**SL**} ∩ {mammals}	346	{CA3} U {hippocampus}
Deep	4541	{Layer 5} U {layer 5–6} U {layer 6} U ({**inner**} ∩ {granule layer}) U ({**inner**} ∩ {plexiform layer}) …	–	–
Granule layer	1296	({**Granule layer**} ∩ {DG}) U ({**stratum granulare**} ∩ {cerebellar cortex}) U ({**stratum granulosum**} U {MOB})	–	–
Fronto-insula	40	{**Fronto-insula**} ∩ {human}	596	{Insula} ∩ {human}
Insula	653	{**Insula**} U {posterior short insular gyrus} U {anterior long insular gyrus} …	349	{Unspecified neocortex}
Posterior horn	33	({**Dorsal horn**} U {lamina III}) ∩ {lumbar}	250	{Unspecified spinal cord}
Ipsilateral projecting	47	{**Ipsilateral-projecting**} ∩ {cerebral cortex}	11,366	{Unspecified pyramidal} U {horizontal} U {inverted} U {extraverted} …
Class III	51	({**Class III**} ∩ {cuticle}) U {VdaD} U …	69	{Unspecified multidendritic-dendritic arborization (DA)}
Mechanosensory	21	{**Touch receptor**}	–	–
Ivy	3	{**Ivy**}	2	{Unspecified Ivy/neurogliaform}

The search terms that has a mapped instance (at the same node) are differentiated in bold from the others that are mapped downstream

### Single hierarchy-based search: use cases of species

The OntoSearch algorithm crawls down the hierarchy for direct hits and crawls up for possible hits. Thus, searching for general concepts will typically generate direct hits, while more specific concepts are more likely to yield possible hits. For instance, querying for ‘rodents’ returns 17,903 neurons as direct hits from the rodentia family, including, ‘rats,’ ‘mice,’ ‘guinea pigs,’ ‘agouti’ and ‘proechimys.’ Searches for ‘vertebrates,’ ‘reptiles,’ ‘primates,’ and ‘nematodes’ similarly identify appropriate data from the respective species and strains per phylogenetic lineage as direct hits. In contrast, querying for ‘spangled skimmer’ (a type of dragon fly) returns 30 neurons from dragon flies of unspecified type as possible hits. Other examples include ‘eastern tiger salamander’ or ‘Philippine long-tailed macaque’ among others. Most searches (e.g., ‘C57BL6 Mouse’) will return both direct and possible hits (2309 and 39, respectively, in this specific case). Additional use cases from this category include searching for neuron types from ‘transgenic mouse’ (encompassing 65 different strains) and ‘knock-in’ or ‘knockout’ mice.

### Multiple hierarchy-based search: use cases of brain regions and cell types

The identification of direct and possible hits in the cases of brain regions has to take into account multiple dimensions when crawling up and down each hierarchy. Consider, for example, a query for ‘stratum moleculare,’ one of the dentate gyrus layers in the hippocampal formation (Fig. 4b). OntoSearch identify the term, its synonyms (e.g., ‘molecular layer’) and its children (inner and outer molecular layer) as direct hits. Furthermore, dentate gyrus neurons that are not reported to reside in disjoint layers (stratum granulosum and hilus) will be returned as possible hits. These neurons might be reported to reside in specific dentate gyrus sub-regions, such as suprapyramidal or crest (Fig. 4b), but these belong to a separate (orthogonal) hierarchy and thus are not disjoint with the target search concept.

As a cell type example, consider the distinct conceptual dimensions commonly used to annotate ‘pyramidal cells’ (corresponding to multiple hierarchies). When querying for ‘ipsilateral-projecting’ pyramidal neurons, OntoSearch returns 47 direct matches and 8951 possible matches. The possible matches include all pyramidal cells that are not annotated with mutually exclusive concepts (thus eliminating ‘callosal-projecting’ neurons), but including other sub-types, such as ‘early-bifurcating’ and ‘late-bifurcating’ or ‘oblique’ and ‘upright.’ Not all cell types are represented with multiple hierarchies: for example, four mutually exclusive types of multidendritic arbor (DA) neurons in the fly larva cuticle are known as ‘class I,’ ‘class II,’ ‘class III,’ and ‘class IV,’ each further divided into sub-types. A query for ‘class III’ returns 51 direct hits and 69 possible hits. The direct hits include all class III sub-types as well as class III neurons of unspecified sub-type. The possible hits include DA neurons of unspecified class as well as fly larva neurons of unspecified type located in the cuticle (or in an unspecified location).

Additional use cases of brain regions and cell types (Table 3) further illustrate synonym and homonym mapping. For instance, ‘Posterior gray horn’ translates into ‘dorsal horn’ of the spinal cord to return 30 direct hits and 100 possible hits. In cell types, the search term ‘mechanosensory’ returns 17 ‘touch receptor’ neurons. In the case of homonyms, the same term corresponds to distinct concepts, as for the ‘granule layer’ of the cerebellum, dentate gyrus, and olfactory bulb, or for the ‘deep layers’ of neocortex and hippocampus. In these cases the OntoSearch logic works differently by querying for multiple concept matches. The direct hits are returned from a union operation on the matching concepts, assuming they are independent from one another. The possible hits, however, are subject to potentially conflicting relationships (e.g., disjoint parents) caused by multiple parent concepts. Therefore, for homonyms OntoSearch does not compute the generalization logic for returning the possible hits. The final example illustrates an OntoSearch hook with the term ‘fronto-insula’ returning 40 direct hits and 596 possible hits. The possible hits only include human insular neurons (eliminating neurons from the ‘rat’ insula), since fronto-insula is exclusively defined in humans.

## Maintenance, continuous development, and future perspectives

Metadata management is a crucial but time-consuming process, which demands a non-trivial long-term plan for sustainability. Organizing the annotation of NeuroMorpho.Org data according to the described ontologies required identifying the conceptual correspondence of each pre-existing entry with a matching level in the appropriate hierarchy. Such painstaking process forced the unequivocal resolution of all terminological ambiguities. This resulted in the *retrospective* correction or stylistic revision of the metadata associated with a substantial proportion of the 37,712 neurons in the database as of v.6.3 (a comprehensive list of the changes is available at NeuroMorpho.Org/WIN.jsp).

In addition to aiding data retrieval via semantic queries, the OntoSearch hierarchies provide a suitable controlled vocabulary for annotating the new neuronal reconstructions continuously deposited in NeuroMorpho.Org while reducing the need for constant expert curation. This *prospective* annotation process was successfully employed to map the metadata of the 12,693 neurons added in v.7.0. Using the ontological indexing system eliminated commonly made mistakes such as introducing a new term for an existing concept. For example, if an article describes the animal strain as ‘Pelophylax esculentus,’ the species ontology resolves this concept as synonymous with ‘edible green frog,’ which is already present in the database.

The knowledge organization system presented here may soon enable the implementation of a web-based service for direct metadata annotation by data producers, gradually reducing the need for database curators. Given the notorious resistance to share data in neuroscience [29, 37], it may seem unrealistic to expect in the foreseeable future that those experimentalists willing to deposit their reconstructions in NeuroMorpho.Org would also agree to map their metadata to formal conceptual ontologies. However, hierarchically structured nomenclatures offer the opportunity to select terms from context-filtered menus, thus facilitating annotation. For instance, when selecting the species, authors might be given a choice of the most common research animals, and when clicking on ‘mouse’ they would be asked to pick only among the relevant strains (see also [38]). After this step (leveraging the OntoSearch ‘hooks’), the dynamically presented brain region menu would exclude neuroanatomical concepts that are irrelevant to the mouse, such as ‘mushroom bodies.’ If selecting neocortex, as a second approximation, annotators would be given a choice between visual, somatosensory, motor, etc. (but not ‘dentate gyrus’ or ‘dorsal horn’ which are concepts only pertaining to hippocampus and spinal cord, respectively). Furthermore, when arriving at the indication of cell type, pyramidal and Martinotti cells would be possibilities, but Purkinje cells and Kenyon cells would not.

A remaining challenge that will continue to prevent the complete removal of the dependence on database curators is that knowledge itself keeps changing with every new publication. While the species taxonomy is relatively well established, the organization of brain regions is still much debated, and the multi-dimensional hierarchies underlying the current version of OntoSearch are destined to evolve. The knowledge about neuron types is even more immature, and a clear community agreement has yet to emerge on a robust classification approach [39]. As information accumulates, expert curation will remain necessary to add new concepts and re-organize existing ones in the OntoSearch framework. At the same time, most concepts in the NeuroMorpho.Org ontology (including animal species, brain regions, etc.) are not specific to neuronal reconstructions. Thus, the same annotation system could be adopted, adapted or expanded by other neuroinformatics initiatives [40], planting the seed for an integrated knowledge base for neuroscience.
